# Nesfatin-1 Receptor: Distribution, Signaling and Increasing Evidence for a G Protein-Coupled Receptor – A Systematic Review

**DOI:** 10.3389/fendo.2021.740174

**Published:** 2021-09-10

**Authors:** Sophia Kristina Rupp, Ellen Wölk, Andreas Stengel

**Affiliations:** ^1^Department of Psychosomatic Medicine and Psychotherapy, University Hospital Tübingen, Tübingen, Germany; ^2^Charité Center for Internal Medicine and Dermatology, Department for Psychosomatic Medicine, Charité - Universitätsmedizin Berlin, Corporate Member of Freie Universität Berlin, Humboldt-Universität zu Berlin and Berlin Institute of Health, Berlin, Germany

**Keywords:** gut-brain axis, food intake, NUCB2, nucleobindin-2, stress, nesfatin-1

## Abstract

**Background:**

Nesfatin-1 is an 82-amino acid polypeptide, cleaved from the 396-amino acid precursor protein nucleobindin-2 (NUCB2) and discovered in 2006 in the rat hypothalamus. In contrast to the growing body of evidence for the pleiotropic effects of the peptide, the receptor mediating these effects and the exact signaling cascades remain still unknown.

**Methods:**

This systematic review was conducted using a search in the Embase, PubMed, and Web of Science databases. The keywords “nesfatin-1” combined with “receptor”, “signaling”, “distribution”, “pathway”, g- protein coupled receptor”, and “binding” were used to identify all relevant articles reporting about potential nesfatin-1 signaling and the assumed mediation *via* a G_i_ protein-coupled receptor.

**Results:**

Finally, 1,147 articles were found, of which 1,077 were excluded in several steps of screening, 70 articles were included in this systematic review. Inclusion criteria were studies investigating nesfatin-1’s putative receptor or signaling cascade, observational preclinical and clinical studies, experimental studies, registry-based studies, cohort studies, population-based studies, and studies in English language. After screening for eligibility, the studies were assigned to the following subtopics and discussed regarding intracellular signaling of nesfatin-1 including the potential receptor mediating these effects and downstream signaling of the peptide.

**Conclusion:**

The present review sheds light on the various effects of nesfatin-1 by influencing several intracellular signaling pathways and downstream cascades, including the peptide’s influence on various hormones and their receptors. These data point towards mediation *via* a G_i_ protein-coupled receptor. Nonetheless, the identification of the nesfatin-1 receptor will enable us to better investigate the exact mediating mechanisms underlying the different effects of the peptide along with the development of agonists and antagonists.

## 1 Introduction

Since the discovery of nesfatin-1 in 2006 in the rat hypothalamus (1), there has been a vast amount of research to further explore the pleiotropic physiological functions of this peptide. Nesfatin-1 is an 82-amino acid polypeptide generated *via* post-translational processing of hypothalamic nucleobindin-2 (NUCB2), a protein of 396 amino acids, whose sequence is highly conserved from fish to mammals (2) pointing towards its physiological relevance. The peptide is predominantly localized in food intake-regulatory nuclei e.g., the arcuate nucleus (Arc), the paraventricular nucleus (PVN) and the nucleus of the solitary tract (3, 4). Accordingly, the peptide became first known for its anorexigenic effects; however, subsequent studies unraveled its many other properties, such as cardiovascular effects, lipid metabolism, reproduction functions, and emotion- related functions (5–7). Subsequently, the peptides’ central part, nesfatin-1_30-59_, which has been identified as the active core of the peptide, was reported to reduce food intake after intracerebroventricular (icv) injection in mice (8, 9) and in rats (10).

However, multiple studies have indicated that nesfatin-1 is also secreted by peripheral tissues such as adipose tissue, gastric mucosa, pancreatic endocrine beta cells and testes and its expression level was found to be 20 times higher in endocrine cells of the oxyntic gastric mucosa than in the brain (11–13).

Numerous studies have reported multifunctional effects of nesfatin-1: peripheral nesfatin-1 affects glucose homeostasis (14) and shows anti-apoptotic and anti-inflammatory effects (15). In addition, peripherally administered nesfatin-1 induces vasoconstriction and elevates blood pressure (16), and moreover decreases antral and duodenal motility (17) and reduces gastric emptying (18). Taken together, the role of nesfatin-1 goes far beyond its initially observed function as a negative modulator of food intake.

In contrast to the growing body of evidence of the pleiotropic peptide’s effects, the receptor mediating these effects is still largely unknown. Although several studies suggest that the peptide activates extracellular and intracellular regulatory pathways involving multiple putative binding sites (19), a specific nesfatin-1 receptor has not yet been identified. Therefore, the present paper was designed to systematically review the findings about nesfatin-1 signaling, especially by focusing on its potential receptor. This systematic review will discuss the state of knowledge on nesfatin-1 signaling, the putative mediation *via* a G_i_ protein-coupled receptor and highlight respective direct and indirect evidence. Lastly, gaps in knowledge will be highlighted to encourage further research hopefully leading to the identification of the nesfatin-1 receptor.

## 2 Methods

We followed the preferred reporting items for systematic reviews and meta-analyses (PRISMA) (20) to report the results of this review.

### 2.1 Search Strategy

This article is a systematic review; articles related to the topic, were searched, collected, and screened. Articles released from the earliest day of publication to the day the search was performed were included. The search was conducted on November 22^nd^ in 2020. We searched Embase, PubMed and Web of Science using the following keywords: “nesfatin-1” combined with “receptor”, “signaling”, “distribution”, “pathway”, G-protein coupled receptor”, and “binding” (**Table 1**). All studies that contained material applicable to the topic were considered. We investigated the reference lists of the included studies to find other potential articles for inclusion. Local experts in the field were consulted for additional studies. Retrieved manuscripts were extracted using a standardized collection tool. Gray literature was not considered due to lack of essential information usually affecting this type of research. There was no date (all studies until November 2020) or species restriction in the search, but the search was limited to texts in English.

**Table 1:** The search strategy of this review.

Search Engines and Databases	Embase PubMed Web of Science
Search date	up to 2020, November, 22
Search terms	Strategy: #1 and #2, #1 AND #3, #1 AND #4, #1 AND #5, #1 AND #6, #1 AND #7 #1 “Nesfatin-1” #2 “Receptor” #3 “Signaling” #4 “Distribution” #5 “Pathway” #6 “G-protein coupled receptor” #7 “Binding”

### 2.2 Inclusion and Exclusion Criteria

The inclusion criteria were as follows: (1) observational preclinical and clinical studies, experimental studies, registry-based studies, cohort studies, population-based studies; (2) Studies that were written in English (3) Studies investigating nesfatin-1 putative receptor or signaling cascade.

The exclusion criteria were as follows: (1) Review articles, surveys, case reports, comments, letters, conference abstracts or posters, and economic evaluation; (2) Studies for which abstracts or full-text articles were not available; (3) Studies that were not available in English; (4) Studies with absence of outcome data.

### 2.3 Study Selection and Data Extraction

One reviewer screened all titles and abstracts retrieved from the electronic searches to identify potentially eligible articles. Full texts of the potentially eligible articles were retrieved. Two reviewers (S.R. and E.W.) screened all full text articles and potentially eligible or unclear full-text articles, determined whether they were eligible or not eligible and recorded the reason for exclusion. Any disagreements between both reviewers were resolved through discussion.

The quality of the studies included in this review was assessed with respect to risk of bias within individual and across studies by thoroughly evaluating the study designs, selection of population/species, methodological procedures applied as well as presentation of the results. Following a full-text review of the eligible studies, one reviewer (E.W.) extracted the relevant data. From each included study the following information was extracted: first author of the publication, year of publication, title, population/species, size of the respective population/species, study type, research question/purpose, and key findings (**Table 2**).

**Table 2:** Main findings of articles (in alphabetical order) included and discussed in this systematic review.

First author	Year	Species	n	Study type	Main findings	Risk of bias
**Angelone T** (21)	2013	Wistar rats	6 rats/group – in total n = 24	Experimental intervention study	By using a nesfatin-1 antibody, the presence of nesfatin-1 was identified in the rat heart. Exogenous nesfatin-1 directly showed negative inotropic and lusitropic effects without affecting coronary motility. These effects were mediated by involving pGC-NPR-A, the cGMP/PKG pathway, and ERK1/2.	
**Ayada C** (22)	2015	Male Wistar rats	7 rats/group – in total n = 28	Experimental intervention study	Nesfatin-1 induced heart failure during clinical treatments by increasing expression of the cardiac L-type Ca^2+^ channel.	Selection bias
**Aydin B** (23)	2018	Male Sprague-Dawley rats	7 rats/group – in total n = 70	Experimental intervention study	Nesfatin-1 elevated mean arterial pressure and modulates heart rate in rats *via* the central cholinergic system.	Selection bias
**Barutcigil A** (24)	2018	Male Wistar rats	Not indicated	Experimental intervention study	Nesfatin-1 dose-dependently induced a relaxation on the endothelium-intact thoracic aorta of rats and produced positive inotropic and chronotropic effects on atria. These effects might be beta-1 receptor independent, while involving the NO-cGMP cascade.	Selection bias
**Brailoiu GC** (25)	2013	Male and female Sprague-Dawley rats (*in vitro*), male Sprague-Dawley rats (*in vivo*), Nucleus ambiguous neurons	6 rats/group (*in vitro*), 5 rats/group (*in vivo*)	Experimental intervention study	Nesfatin-1 increased cytosolic Ca^2+^ levels *via* a G_i/o_-coupled mechanism in cardiac vagal neurons of nucleus ambiguous by involving P/Q type voltage-activated Ca^2+^ channels. Moreover, nesfatin-1 led to a dose-dependent depolarization of cardiac vagal neurons *via* a G_i/o_-coupled mechanism.	Selection bias
**Brailoiu GC** (26)	2007	Hypothalamic neurons, male and female Sprague-Dawley rats	170 neurons, different group sizes, number of rats not indicated	Experimental intervention study	In rats nesfatin-1 was present in hypothalamic and brainstem neurons and stimulated Ca^2+^ influx *via* GPCR.	
**Buzcu H** (27)	2019	Female Sprague-Dawley rats	8 rats/group – in total n = 56	Experimental intervention study	In acute pancreatitis nesfatin-1 showed an antioxidant and anti-inflammatory effect *via* the melanocortin signaling pathway.	Selection bias
**Chen X** (28)	2015	Male C57BL/6 mice, male Wistar rats	6 rats/group – in total n = 12, 10 mice/group – in total n = 30	Experimental intervention study	Nesfatin-1 reduced dark-phase food intake in mice by inhibiting excitability of dopaminergic neurons in the VTA and reducing dopamine release in the nucleus acumbens.	Selection bias
**Chen Z** (29)	2018	Human neuroblastoma SH-SY5Y cells	4 cells/group – in total n = 24	Experimental intervention study	In human neuroblastoma SH-SY5Y cells nesfatin-1 led to an over-expression of synapsin I and phosphorylated ERK1/2 mediated *via* CRF_1_.	
**Dong J** (30)	2013	Male Kumming SPF mice	Not indicated	Experimental intervention study	Nesfatin-1 normalized free fatty acids and was thus able to improve lipid disorder *via* activation of AMPK-ACC pathway in T2DM mice.	Selection bias
**Dore R** (31)	2017	Male Wistar rats, male, C57BL/6N wild type mice	Different group sizes (5-10/group), 6 groups in total	Experimental intervention study	Nesfatin-1 treatment increased dry heat loss, iBAT and tail temperature through activation of the melanocortin system.	Selection bias
**Erfani S** (32)	2018	Male Wistar rats	7 rats/group – in total n = 28	Experimental intervention study	Nesfatin-1 had neuroprotective effects in neuronal cells and neuroinflammatory processes caused by brain ischemia by decreasing activation of caspase-3.	Selection bias
**Fan XT** (33)	2018	Male C57BL/6J mice (GHSR+/+) and (GHSR-/-)	6-8 GHSR+/+ mice/group – in total n = 38, 3-5 GHSR-/-mice/group – in total n = 17	Experimental intervention study	Nesfatin-1 required GHSR for mediating its effects on food intake and glucose metabolism.	Selection bias
**Feijóo-Bandín S** (34)	2013	humans, male Sprague-Dawley rats, HL-1 cardiac muscle cells of mice	178 men, 90 women, 44 rats, number of muscle cells not indicated, different group sizes	Experimental intervention study	Cardiomyocytes can synthesize and release nesfatin-1 and the peptide stimulated glucose uptake by HL-1 cells and cardiomyocytes and translocation of GLUT4 to the periphery of these cells.	Selection bias
**Feng H** (35)	2017	Male Wistar rats	144 rats, group sizes not indicated	Experimental intervention study	Nesfatin-1-expressing neurons in the hippocampus project to the VMH and there, nesfatin-1 modulated GD-responsive neurons and thus had an impact on the control of gastrointestinal functions.	Selection bias
**Gao S** (36)	2016	Sprague-Dawley rats	Not indicated	Experimental intervention study	Nesfatin-1 altered firing rates of GD-responsive VMH neurons, thereby inhibiting food intake, gastric acid production, gastric motility, and gastric emptying.	
**Ge JF** (37)	2015	Male Sprague-Dawley rats	10 rats/group – in total n = 40	Experimental intervention study	Nesfatin-1 mediated anxiety-like behavior in rats without altering memory.	Selection bias
**Guo FF** (38)	2015	Male Wistar rats	Not indicated	Experimental intervention study	Nesfatin-1 acted as an inhibitory neurotransmitter to regulate gastric motility *via* the LHA-PVN pathway.	Selection bias
**Heidarza-deh H** (39)	2018	Male meat-type chicken	44 chicken/group – in total n = 304	Experimental intervention study	In neonatal chicks nesfatin-1 used CRF1/CRF2 as well as H1-R and H3-R to mediate its anorexigenic effect.	Selection bias
**Ishida E** (40)	2012	mouse neuroblastoma cell line NB41A3, male C57/BL6 (B6) mice	Not indicated	Observational study	Nesfatin-1 bound to cell surface of NB41A3 cells and mouse hypothalamus indicating the presence of a specific nesfatin-1 receptor. Moreover, nesfatin-1 induced phosphorylation of CREB *via* binding to a Gi/o protein-coupled receptor and by utilizing Ca^2+^ influx and/or MAPK signaling cascade.	Selection bias
**Iwasaki Y** (41)	2009	Male ICR mice	Not indicated	Experimental intervention study	Peripheral nesfatin-1 stimulated Ca^2+^ influx through voltage-gated N-type channels, thereby directly activating afferent vagal neurons.	Selection bias
**Jia FY** (42)	2013	Male Sprague-Dawley rats	9 rats/group – in total n = 36	Experimental intervention study	Nesfatin-1 was involved in CRF/CRF1 signaling pathways in the brain, contributing to visceral hypersensitivity in rats.	Selection bias
**Jiang L** (43)	2020	human neuroblastoma SH-SY5Y cells, Sprague-Dawley rats	5 human samples, 15 rats/group – in total n = 45	Experimental intervention study	Treatment with nesfatin-1 obviated cartilage degeneration in rats which plays a major role in the development of osteoarthritis.	
**Kan JY** (44)	2016	humans, male inbred mice (BALB/cByJNarl)	119 healthy donors, 160 colon cancer patients – in total n = 279 subjects, number of mice not indicated	Experimental intervention study	Nesfatin-1/NUCB2 increased invasion, migration and mesenchymal phenotype in colon cancer *via* LKB1/AMPK/TORC1/ZEB1 signaling pathways and may be a prospective marker for prediction of metastasis.	Selection bias
**Kerbel B** (45)	2012	goldfish Carassius auratus	n = 36	Experimental intervention study	There is a possible relationship between nesfatin-1 and ghrelin, CCK and orexin A in goldfish to regulate food intake.	
**Levata L** (46)	2019	Male C57BL/6J mice	Not indicated	Experimental intervention study	Nesfatin-1 increased peripheral sympathetic outflow, resulting in iBAT thermogenesis and body weight loss.	Selection bias
**Li C** (47)	2014	Wistar rats	Not indicated	Experimental intervention study	In the substantia nigra nesfatin-1 post-synaptically hyperpolarized dopaminergic neurons, thus leading to a direct inhibition of these neurons.	
**Li T** (48)	2021	human HTR-8/trophoblasts	Not indicated	Experimental intervention study	Overexpression of nesfatin-1 increased human trophoblast proliferation, migration, invasion, and epithelial-mesenchymal transition and simultaneously suppressed oxidative stress.	
**Li ZL** (49)	2013	Male Wistar rats	n = 348 in different experiments	Experimental intervention study	Nesfatin-1 modulated gastrointestinal motility by affecting ghrelin-responsive GD neurons in the arcuate nucleus in rats.	Selection bias
**Li Z** (14)	2013	Male C57BL/6J mice, HFD- induced obese mice, Sprague-Dawley rats	Approximately n = 44 mice, number of rats not indicated	Experimental intervention study	Peripheral nesfatin-1 administration altered glucose metabolism in mice *via* increasing insulin secretion and insulin sensitivity by altering AKT phosphorylation and GLUT 4 membrane translocation in the adipose tissue, liver and skeletal muscle.	
**Lu QB** (50)	2018	Wistar-Kyoto rats, spontaneous hypertensive rats (SHR), human VSMCs, rat VSMCs	6 Wistar-Kyoto rats/group – in total n = 84, 6 SHR/group – in total n = 68	Experimental intervention study	Nesfatin-1 promoted VSMC differentiation and proliferation, leading to hypertension and vascular remodeling.	
**Maejima Y** (51)	2017	Male C57BL/6J mice, HEK239 cells	16 mice/group – in total n = 35	Experimental intervention study	Nesfatin-1/NUCB2 expression was found in pancreatic beta-cells in mice. Here, nesfatin-1 was found to inhibit Kv-channels in a direct manner.	Selection bias
**Maejima Y** (52)	2009	Male Wistar rats, Zucker-lean rats, Zucker-fatty rats	Not indicated	Experimental intervention study	Nesfatin-1 induced anorexia in a leptin-independent, but melanocortin-dependent manner *via* oxytocin neurons in the PVN.	
**Mazza R** (53)	2015	goldfish Carassius auratus	4 goldfish/group – in total n = 44	Experimental intervention study	Exposure of the isolated and perfused working heart to nesfatin-1 resulted in positive inotropism.	
**Mori Y** (54)	2019	Male C57BL/6J mice, transgene nucleobindin-2 mice, human VECs and VSMCs	n = 25 C57BL/6J mice, n = 13 transgene mice, different group sizes	Experimental intervention study	Nesfatin-1 administration dose-dependently suppressed peripheral artery remodeling in vascular endothelial cells and decreased neointimal hyperplasia.	Selection bias
**Nair N** (55)	2016	Male and female zebrafish (Danio rerio	8 zebrafish/group – in total n = 64	Experimental intervention study	NUCB2/nesfatin-1 may be localized in cardiomyocytes in zebrafish and administration of nesfatin-1 led to inhibition of end diastolic and end systolic volumes, decreasing heart rate and cardiac output.	
**Nakata M** (56)	2011	Male ICR mice	Not indicated	Experimental intervention study	Nesfatin-1 dose-dependently stimulated both insulin secretion in islets and intracellular Ca^2+^ levels in beta-cells under elevated plasma glucose concentration.	Selection bias
**Oh I** (1)	2006	Zucker obese rats, male Wistar rats	n = 4-10 rats/group, exact number of rats not indicated	Experimental intervention study	Nesfatin-1-induced satiety was associated with leptin-independent melanocortin signaling in the hypothalamus.	Selection bias
**Osaki A** (57)	2014	Male ICR mice	3 mice/group – in total n = 18	Experimental intervention study	Nesfatin-1 plays a physiological role in regulating blood pressure in mice by altering vascular contractility.	Selection bias
**Ozcan M** (58)	2016	Wistar rats	Not indicated	Experimental intervention study	Nesfatin-1 interacted with a GPCR and used a PKC-dependent mechanism to induce calcium influx in neonatal rat dorsal root ganglion neurons.	
**Ozturk CC** (59)	2015	Male Sprague-Dawley rats	n = 48 rats, group sizes not indicated	Experimental intervention study	The anti-inflammatory effects of nesfatin-1 in colitis were mediated *via* oxytocin and ghrelin receptors.	Selection bias
**Price CJ** (60)	2008	Male Sprague-Dawley rats	Number of rats not indicated, 85 neurons	Experimental intervention study	In the PVN nesfatin-1 regulated the membrane potential of different subtypes of neurons.	Selection bias
**Price CJ** (61)	2008	Male Sprague-Dawley rats	Number of rats not indicated, 102 neurons	Experimental intervention study	Nesfatin-1 exposure led to hyperpolarization in NPY-expressing neurons in the arcuate nucleus. These effects might be mediated *via* KATP channels.	Selection bias
**Prinz P** (62)	2016	Male Sprague-Dawley rats	n = 6 rats	Observational study	Bound nesfatin-1 radiolabel was detected in various peripheral organs and several brain nuclei.	
**Ramanja-neya M** (63)	2015	Female Wistar rats, human H295R adrenal cortex cells, mouse Y1 tumor cells	Not indicated	Experimental intervention study	Nesfatin-1 administration suppressed adrenocortical cell growth while increasing apoptosis.	Selection bias
**Ranjan A** (64)	2019	Male Parks strain mice	n = 21 mice	Experimental intervention study	Nesfatin-1 in mouse testes led to an increase in testosterone production, which was accompanied by higher expression of steroidogenic enzymes and insulin receptor protein.	
**Ranjan A** (65)	2019	Male Parks strain mice	n = 27 mice	Experimental intervention study	Nesfatin-1 played a role in spermatogenesis and steroidogenesis of prepubertal mice by direct action on the testis in association with the progression to puberty.	
**Ranjan A** (66)	2020	Male Parks strain mice	10-20 mice/group – in total n = 50	Experimental intervention study	Nesfatin-1 played a crucial role in ameliorating the testicular functions of T2DM mice by altering the circulating lipid profile.	
**Shen XL** (67)	2017	Male C57BL/6J mice, MES23.5 cells	6 mice/group – in total n = 30	Experimental intervention study	Nesfatin-1 showed a neuroprotective effect in dopaminergic neurons by protecting against MPP+/MPTP-induced neurotoxicity. These effects might be mediated *via* activation of the C-Raf-ERK1/2 signaling cascade.	Selection bias
**Shimizu H** (9)	2009	Male ICR mice, db/db mice	Not indicated	Experimental intervention study	The middle segment of nesfatin-1 caused anorexia *via* a leptin-independent mechanism.	
**Stengel A** (18)	2009	Male Sprague-Dawley rats	Not indicated	Experimental intervention study	Nesfatin-1 in rats led to a delayed inhibition of food intake in the dark phase, involving CRF2 receptor-dependent pathways.	Selection bias
**Tan J** (68)	2016	HGSMC cells	20,000 cells/group – in total n = 60,000	Experimental intervention study	Nesfatin-1 inhibited HGSMC viability and adhesion.	
**Tanida M** (69)	2015	Male Wistar rats, Zucker fatty rats, HFD rats	7 Wistar rats/group – in total n = 28, 5-6 Zucker fatty rats/group – in total n = 20-22, 5-6 HFD rats/group – in total n = 20-22	Experimental intervention study	Hypothalamic ERK signaling underlain the sympathoexcitatory effect of nesfatin-1 on energy intake and lipid metabolism.	
**Tanida M** (70)	2011	Male Wistar rats	4-10 rats/group – in total n = 28	Experimental intervention study	Nesfatin-1 modulated central sympathetic outflow, thereby stimulating renal sympathetic outflow and increasing blood pressure.	Selection bias
**Tasatargil A** (71)	2017	Male Wistar rats	8 rats/group – in total n = 32	Experimental intervention study	Nesfatin-1 showed cardioprotective effects in rats by decreasing myocardial apoptosis and inflammation which in turn reduces myocardial infarct size.	Selection bias
**Vélez EJ** (72)	2020	rat GH3 cells, RC-4B/C cells	Not indicated	Experimental intervention study	Nesfatin-1 and NLP showed a direct effect on somatotrophs by downregulating the synthesis of GH *via* a GPCR through the AC/PKA/CREB signaling pathway, most likely including a G-α-inhibitory subunit.	
**Wang Q** (73)	2014	Male Wistar rats	65-65 rats/experiment – in total n = 246	Experimental intervention study	In the central nucleus of the amygdala nesfatin-1 modulated the activity of GD-sensitive neurons and gastric motility.	Selection bias
**Wu D** (74)	2014	Male Sprague-Dawley rats	51 rats/group – in total n = 102	Experimental intervention study	Hypothalamic nesfatin-1 was involved in the regulation of glucose homeostasis and hepatic insulin sensitivity, associated with the activation of the mTOR-STAT3 signaling pathway.	Selection bias
**Xia ZF** (75)	2012	Male Sprague-Dawley rats, vagal neurons of Sprague-Dawley rats	Not indicated	Experimental intervention study	Nesfatin-1 inhibited gastric acid secretion stimulated by a central vagal mechanism in rats involving T-Typ Ca^2+^ channels.	Selection bias
**Xu L** (76)	2017	Male Wistar rats	46-120 rats/experiment – in total approximately 439	Experimental intervention study	Nesfatin-1 signaling in the lateral hypothalamic area modulated the activation of GD-responsive neurons, gastric motility and gastric secretion also involving melanin-concentrating hormone signaling.	Selection bias
**Xu L** (77)	2015	Male Wistar rats	6-58 rats/experiment – in total n = 262	Experimental intervention study	Nesfatin-1 administration into the BMA increased firing rate of GD-excitatory neurons, while decreasing firing rates of GD-inhibitory neurons. Nesfatin-1 in the BMA is involved in decreasing gastric motility and the Arc may also play a role in this regulating process.	Selection bias
**Yamawa-ki H** (16)	2012	Male Wistar rats	8-23 rats/experiment – in total n = 58	Experimental intervention study	Nesfatin-1 modulated peripheral arteria contractility by impairing cGMP release, thus inhibiting the SNP-induced smooth muscle relaxation.	Selection bias
**Yang M** (78)	2012	Male Sprague-Dawley rats	8-40 rats/experiment – in total n = 68	Experimental intervention study	Icv injection of nesfatin-1 increased peripheral and hepatic insulin sensitivity by decreasing gluconeogenesis and promoting peripheral glucose uptake through AMPK/AKT/TORC2 pathway.	Selection bias
**Yin Y** (79)	2015	C57BL/6J mice	24-30 mice/experiment, exact number of mice not indicated	Experimental intervention study	Nesfatin-1 modulated lipid accumulation in hepatocytes *via* an AMPK-dependent mechanism.	
**Ying J** (80)	2015	Male Wistar rats	Not indicated	Experimental intervention study	Nesfatin-1 inhibited L-type Ca^2+^ channels *via* the MC4-R and involved the Gβγ subunit of Gi/o-protein and the downstream PKCθ pathway.	Selection bias
**Yosten GL** (81)	2014	Male Sprague-Dawley rats	5-13 rats/group – in total n = 248	Experimental intervention study	The hypertensive effect of nesfatin-1 may require both activation of oxytocinergic neurons and recruitment of CRF neurons.	Selection bias
**Yuan JH** (82)	2017	Male Wistar rats	24-56 rats/experiment, exact number of rats not indicated	Experimental intervention study	Nesfatin-1 played a role in inhibition of food intake, alteration of the excitability of glucose sensitive neurons in the LPBN and an increase of UCP expression in brown adipose tissue by involving the melanocortin system.	Selection bias
**Zhang JR** (83)	2018	Male Sprague-Dawley rats	6 rats/group, exact number of rats not indicated	Experimental intervention study	Nesfatin-1 stimulated VSMC proliferation, migration, and phenotype switch from a contractile to a synthetic state.	Selection bias
**Zhang T** (84)	2019	Male Sprague-Dawley rats	6 rats/group – in total n = 54	Experimental intervention study	Hypothalamic nesfatin-1 regulated feeding behavior through the MC3/4R-ERK signaling pathway.	
**Zhang X** (85)	2018	Siberian sturgeons	6-8 sturgeons/group – in total n = 45	Experimental intervention study	Nesfatin-1 reduced food intake in Siberia sturgeon predominantly *via* the CCK-CCK1R signaling pathway.	
**Zhou XP** (86)	2016	Male Sprague-Dawley rats	15-18 rats/group, exact number of rats not indicated	Experimental intervention study	Nesfatin-1/NUCB2 in the amygdala was involved in the pathophysiology of IBS-like visceral hypersensitivity, likely by involving glucocorticoid and mineral corticoid receptor pathways.	Selection bias

AC, adenylyl cyclase; AMPK, 5’ AMP-activated protein kinase; BMA, basomedial amygdala; CCK, cholecystokinin; CCK1-R, cholecystokinin1 receptor; cGMP, cyclic guanosine monophosphate; CREB, cAMP response element-binding protein; CRF, corticotropin-releasing factor; CRF_1_, corticotropin-releasing factor receptor 1; EGF, Epidermal growth factor; ERK, extracellular signal-regulated kinases; GD, gastric distension; GHSR, growth hormone secretagogue receptor; GLUT4, glucose transporter type 4; GPCR, G protein-coupled receptor; H1-R, histamine receptor 1; H3-R, histamine receptor 3; HFD, high-fat diet; HGSMC, human gastrointestinal smooth muscle cells; iBAT, interscapular brown adipose tissue; IBS, irritable bowel syndrome; icv, intracerebroventricular; KATP channel, ATP-sensitive potassium channel; Kv, voltage-gated potassium; LHA, lateral hypothalamic area; LKB1, liver kinase B1; LPBN, lateral parabrachial nucleus; MAP, mean arterial pressure; MAPK, mitogen-activated protein kinase; MC3-R, melanocortin 3 receptor; MC4-R, melanocortin 4 receptor; MEK, MAPK kinase/ERK kinase; mTOR, mammalian target of rapamycin; NLP, nesfatin-1-like peptide; NO, nitric oxide; NPR-A, natriuretic peptide receptor A; NPY, neuropeptide Y; NUCB2, nucleobindin-2; pGC, particulate guanylate cyclase; PKA, protein kinase A; PKC, protein kinase C; PKCθ, protein kinase C theta; PKG, protein kinase G; PVN, paraventricular nucleus; RCC, renal cell carcinoma; SNP, sodium nitroprusside; SPF, Specific-pathogen-free; STAT, signal transducers and activators of transcription; TORC1, target of Rapamycin complex 1; TORC2, target of Rapamycin complex 2; TRH, thyrotropin-releasing hormone; T2DM, type 2 diabetes mellitus; UCP, uncoupling protein; VEC, Vascular endothelial cells; VMH, ventromedial hypothalamus; VSMC, vascular smooth muscle cells; ZEB1, zinc finger E-box-binding homeobox 1.

## 3 Results

**Figure 1:**
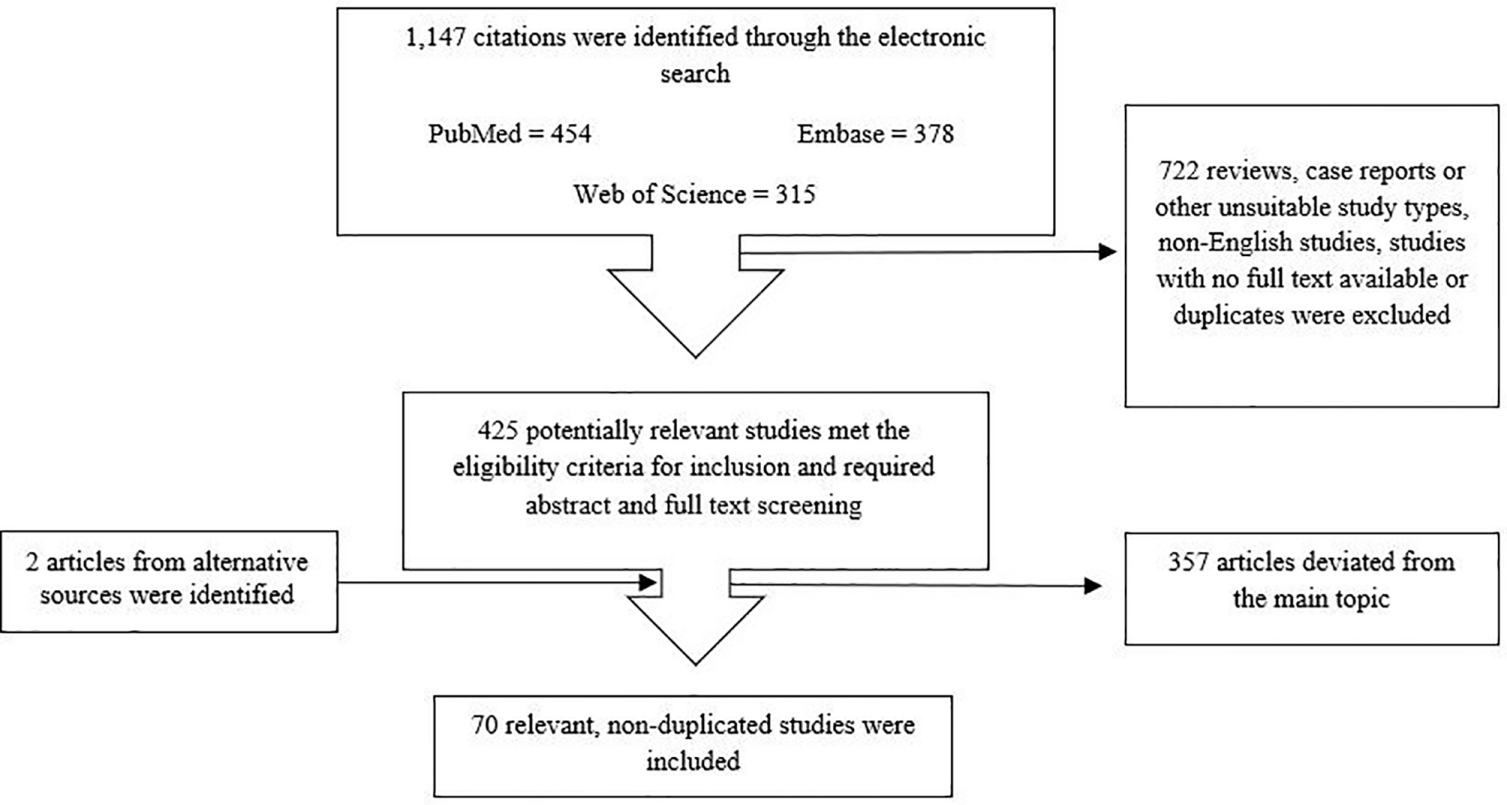
Flowchart for article screening and selection.

1,147 articles were identified after searching the databases using the keywords mentioned above. Reviews, surveys, case reports, comments, letters, conference abstracts or posters, as well as economic evaluation and non-English language studies or studies with no full text available, and duplicates were excluded. This deceased the number to 425 articles. Next, title and abstract were screened and studies which deviated from the main topic were excluded; thus, 128 studies required full-text screening. Here, studies not related to the review topic were excluded. Ultimately, 70 articles were selected for this systematic review. The PRISMA flow diagram schematically depicts the article selection process (**Figure 1**). **Table 2** shows the main results of these articles.

### 3.1 Quality Assessment

The current review consists of observational preclinical and clinical studies or experimental studies, assuming a risk for bias. There are several issues that may contribute to limited comparability between studies and thus limited transferability of study results in general. First, the studies included different species or population, which may lead to limited comparability. Second, the respective study populations differed in sample size, while the study methods, treatments and observation periods used were also very heterogeneous. Furthermore, we only reviewed the abstracts and full texts published in English. Studies in other languages have not been included. Additionally, we searched only three databases for potentially eligible studies. Taken together, bias cannot be ruled out for the studies included in this systematic review; thus, these limitations should be kept in mind when interpreting the results discussed here.

## 4 Discussion

**Figure 2:**
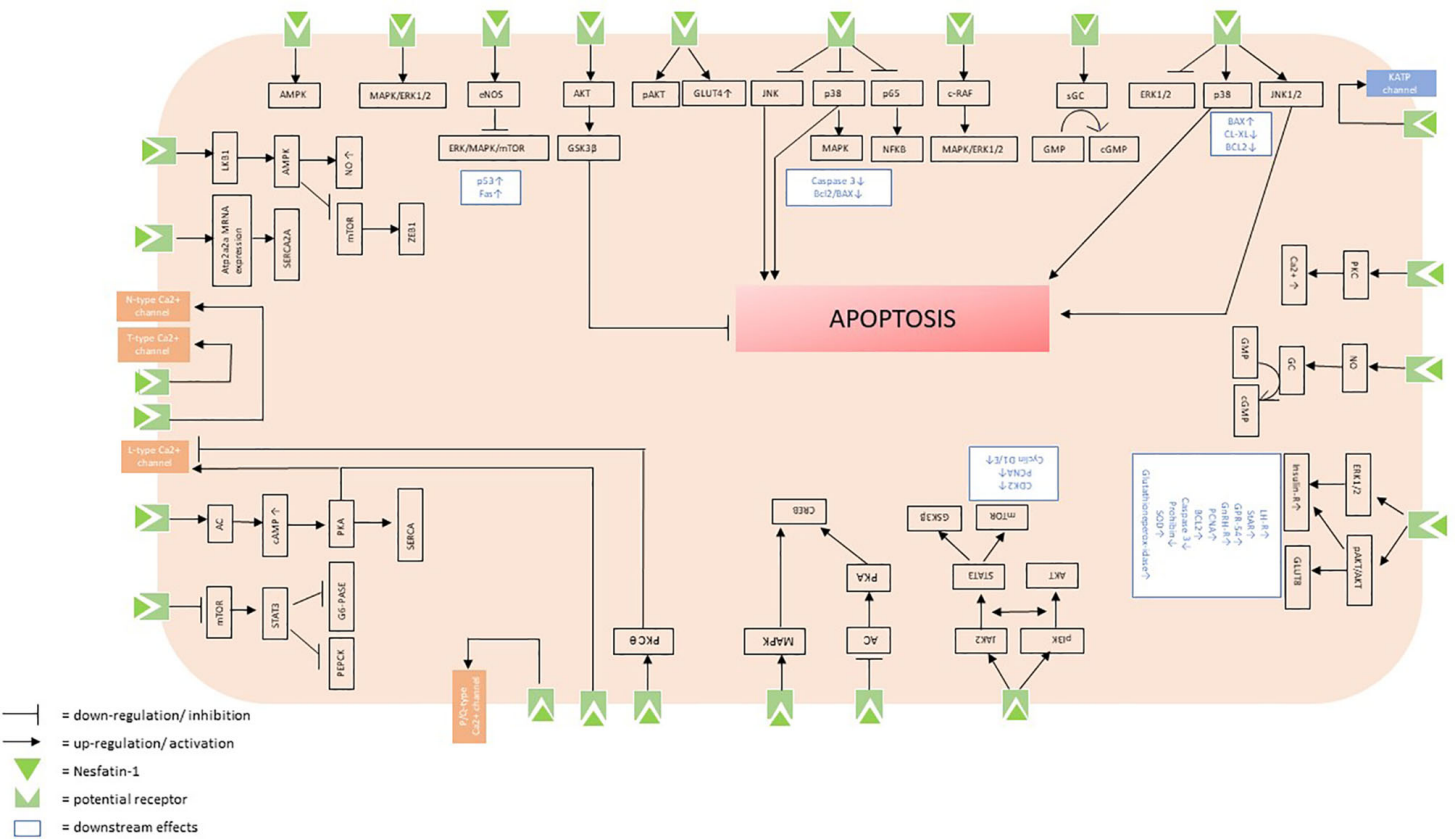
Putative intracellular signaling cascades initiated by the binding of nesfatin-1 to its receptor.

The aim of this systematic review was to identify and summarize the state of knowledge on signaling cascades of nesfatin-1 and to shed light on the peptide’s potential receptor. Nesfatin-1 affects multiple sites in the organism and elicits a variety of intracellular effects, which accounts for the pleiotropic nature of this peptide. The multiple intracellular signaling cascades triggered by nesfatin-1 (**Figure 2**) only reinforce the multifaceted nature of this peptide and highlight that it may be of great importance to understand the exact cascades by further investigating the particular type and localization of the potential receptor in the future.

### 4.1 Intracellular Signaling and Potential Receptor

By using autoradiography binding of radiolabeled nesfatin-1 in the gastric mucosa of corpus and antrum, in duodenum, jejunum and ileum and centrally in the cortex, paraventricular nucleus of the hypothalamus, area postrema, dorsal motor nucleus of the vagus nerve and cerebellum was shown, giving rise to respective expression sited of the receptor (62). Since nesfatin-1 is known to be able to cross the blood-brain barrier, peripheral nesfatin-1 might access and then bind to these central receptors (87). In addition, NUCB2/Nesfatin-1 was found to be widely distributed in the central nervous system of mice (88) and rats (3, 89).

#### 4.1.1 AMPK Signaling Pathway

Converging evidence points towards a mediation of nesfatin-1’s effects *via* a G-protein coupled receptor: Previous results suggest the presence of a nesfatin-1 receptor in hepatocytes whose activation stimulates the phosphorylation of AMPK, reducing hepatic lipid accumulation. These alterations were associated with a significant attenuation of peroxisome proliferator-activated receptor γ (PPARγ) and sterol-regulatory element-binding protein-1 (SREBP1), which are known lipogenesis-related transcription factors (79). Furthermore, in diabetic mice low-dose injection of nesfatin-1 also regulated fatty acid metabolism *via* activation of an AMPK-ACC pathway (30). The assumption of an influence of nesfatin-1 on AMPK signaling cascades is consistent with recent findings, highlighting that nesfatin-1/NUCB2 enhances invasion, migration, and mesenchymal phenotype in colon cancer *via* liver kinase B1 (LKB1)/AMPK/target of rapamycin kinase complex I (TORC1)/ZEB1 signaling pathways (44). In line with this, it was further shown that nesfatin-1 suppresses peripheral artery remodeling likely by increasing NO production and LKB1-mediated activation of AMPK in vascular endothelial cells in mice (54). However, in the testis of mice nesfatin-1-induces an increase in G protein-coupled receptor 54 (GPR-54) accompanied by increases in PCNA, Bcl2, androgen receptor (AR), GLUT8, insulin receptor and gonadotropin-releasing hormone receptor (GnRH-R) (64, 65). In addition, a potential role of protein kinase B (AKT) as a signaling mechanism for nesfatin-1-induced glucose uptake and activation of the AMP-activated protein kinase (AMPK)/extracellular signal-regulated kinase 1/2 (ERK1/2) system was found through which nesfatin-1 enhances insulin sensitivity in the testis of mice (64, 66). Supporting these findings, icv injection of nesfatin-1 increased peripheral and hepatic insulin sensitivity by decreasing gluconeogenesis and promoting peripheral glucose uptake through the AMPK/AKT/TORC2 pathway (78).

#### 4.1.2 MAPK Signaling Pathway

Furthermore, previous results assumed the presence of a nesfatin-1-specific-receptor, likely a Gi/o protein-coupled receptor, on the cell surface of a murine neuroblastoma cell line (NB41A3 cells) and mouse hypothalamus. Here, binding of nesfatin-1 activates the cAMP-response reporter (CRE) and further phosphorylates CREB in these cells by utilizing Ca^2+^ influx and/or the MAPK signaling pathway (40). Other studies also point to a crucial role of nesfatin-1 in the ERK/MAPK/mechanistic Target of Rapamycin (mTOR) signaling pathway by upregulating endothelial NO synthase (eNOS) levels in human gastrointestinal smooth muscle cells (HGSMC). Here, nesfatin-1 increases pro-apoptotic factors p53 and Fas, thereby accelerating apoptosis in HGSMC. Furthermore, nesfatin-1 led to reduced expression of ERK1/2, p38, MAPK and mTOR in HGSMC (68). Moreover, in adrenocortical cells nesfatin-1 increases apoptosis by involving ERK1/2, p38, c-Jun N-terminal kinase 1/2 (JNK1/2) signaling pathways, Bcl-2-associated X protein (Bax), B-cell lymphoma-extra-large (BCL-XL), and Bcl-2 genes (63). In accordance, nesfatin-1 decreased apoptosis rate in rat chondrocytes also through the Bax/Bcl-2 signaling pathway and further through suppressing both NF-κB and MAPK signaling pathways (43). Furthermore, it was found that nesfatin-1 ameliorates levels of human gene for type H procollagen (CoI2a1) while it decreases the IL-1beta induced expression of matrix metalloproteinases (MMP), a disintegrin and metalloproteinase with thrombospondin motifs 5 (ADAMTS5), cyclooxygenase 2 (COX-2), caspase-3, nitric oxide (NO), inducible nitric oxide synthase (iNOS), prostaglandin E2 (PGE2), and interleukin-6. The nesfatin-1-induced decrease of caspase-3 activity was also detected in the hippocampus accompanied by fewer ionized calcium binding adaptor molecule 1 (Iba-1)-positive cells (32). Interestingly, consecutive intraperitoneal (ip) administration of nesfatin-1 for three weeks decreases brain-derived neurotrophic factor (BDNF) and phosphorylated-ERK levels in the hippocampus and prefrontal cortex (PFC), thereby downregulating the ERK signaling pathway (37). In accordance, it was demonstrated that nesfatin-1 protects dopaminergic neurons from 1-Methyl-4-phenylpyridinium (MPP+)- and 1-Methyl-4-phenyl-1,2,3,6-tetrahydropyridin (MPTP)-induced neurotoxicity both *in vivo* and *in vitro*, probably *via* activation of the C-Raf/ERK1/2 signaling cascade (67). Regarding nesfatin-1’s influence on the ERK signaling cascade, it was further found that the peptide regulates the autonomic nervous system through ERK signaling in corticotropin-releasing factor (CRF) positive neurons in the paraventricular nucleus (PVN). Precisely, central nesfatin-1 administration stimulated MAPK activity thereby enhancing ERK1/2 phosphorylation in CRF neurons of the PVN (69). Consistent with these findings, also in human neuroblastoma SH-SY5Y cells, it was observed that the stimulatory effects of nesfatin-1 on synapsin I expression are mediated by CRF_1_ through the cAMP/MAPK/ERK pathway (29).

#### 4.1.3 CRF Signaling Pathway

Regarding nesfatin-1’s influence on CRF signaling pathways, gastric distension (GD)-responsive neurons were found in ventromedial hypothalamic nucleus (VMH), whose firing rate was altered by nesfatin-1, most likely through interactions with CRF signaling pathways (35). This hypothesis was strengthened by the observed association of nesfatin-1 and irritable bowel syndrome-like visceral hypersensitivity that is also mediated, at least in part, by CRF/CRF_1_ signaling pathways in the brain (42). In consistence with these findings, it was reported that nesfatin-1 reduced dark-phase food intake *via* CRF pathways (18). Furthermore, in neonatal chicks nesfatin-1 used CRF_1_/CRF_2_ as well as histamine (H)_1_ and H_3_ receptors to mediate its anorexigenic effect. Interestingly, co-injection of nesfatin-1 and chlorpheniramine (H_1_ antagonist) blunted nesfatin-1-induced hypophagia, while co-administration with thioperamide (H_3_ antagonist) reinforced the hypophagic effect of nesfatin-1. This divergence in the effects of H_1_ and H_3_ receptors can be explained by the fact that H_1_ is a postsynaptic receptor, while H_3_ is a presynaptic autoreceptor, leading to the hypothesis that H_1_ and H_3_ play a different regulatory role in the feeding behavior influenced by nesfatin-1 (39).

#### 4.1.4 Melanocortin Signaling Pathway

Using the melanocortin signaling pathway, nesfatin-1 was found to alter firing rates of GD-responsive neurons in the VMH (36), baso-medial amygdala (BMA) (77), arcuate nucleus (49) and central nucleus of the amygdala (73). Also, by involving the melanocortin system nesfatin-1 alters the excitability of glucose sensitive neurons in the lateral parabrachial nucleus (LPBN) and increases the level of uncoupling protein (UCP) expression in brown adipose tissue (BAT) (82). In accordance, it was assumed that nesfatin-1-induced satiety is associated with leptin-independent melanocortin signaling in the hypothalamus (1). This hypothesis was strengthened by a study showing that hypothalamic nesfatin-1 regulates feeding behavior through the MC_3/4_-ERK signaling pathway. Interestingly, in this study, hypothalamic nesfatin-1 mediates its effects *via* MC_3/4_, while not altering expression of MC_3/4_ (84). Furthermore, through activation of the melanocortin system icv nesfatin-1 treatment increases dry heat loss, interscapular (i) BAT and tail temperature. Moreover, nesfatin-1 administration upregulated the expression of POMC and MC_3_ mRNA in the hypothalamus along with an elevation of iodothyronine deiodinase 2 (Dio2), UCP1 and PPARγ-1 alpha mRNA in the iBAT (31). Furthermore, the nesfatin-1-induced elevation in the mRNA expression of the cAMP-responsive gene Dio2 strengthens the hypothesis of an activation of the β-adrenergic/cAMP signaling pathway, which is in line with other findings emphasizing the established role of nesfatin-1 in sympathetic nerve activity by activating the central melanocortin system (70). On the other hand, only the midsegment of nesfatin-1 proves effective and leads to a higher activation of c-Fos in the brainstem nucleus of the solitary tract (NTS). Furthermore, administration of the middle segment of nesfatin-1 results in an increase in the expression of proopiomelanocortin (POMC), cocaine- and amphetamine-regulated transcript (CART) mRNA in the NTS (9). In adult ventricular myocytes nesfatin-1 targets MC_4_, sequentially coupling to the G_βγ_ subunits of G_i/o_, leading to the subsequent activation of the novel protein kinase C (PKC) θ isoform, subsequently resulting in an inhibition of L-Type Ca^2+^ channels associated with a hyperpolarizing shift in the voltage-dependence of inactivation. Notable the nesfatin-1 mediated inhibition of calcium-influx was not affected by Kt-5720, a PKA antagonist indicating that the cAMP/PKA pathway is not involved in the nesfatin-1-induced L-Type Ca^2+^ channel response in ventricular myocytes (80). In contrast, it was observed that both nesfatin-1 and nesfatin-1-like peptide directly affect somatotrophs *via* binding to a GPCR containing a G-α-i subunit and utilizing the adenylyl cyclase (AC)/protein kinase A (PKA)/CREB signaling pathway subunit, thereby downregulating the synthesis of ghrelin hormone (72).

#### 4.1.5 Ion Currents

However, in cardiac vagal neurons nesfatin-1 may increase cytosolic Ca^2+^ levels *via* a G_i/o_-coupled mechanism by involving P/Q-type voltage-activated Ca^2+^ channels (25), while in hypothalamic neurons the peptide might mediate Ca^2+^ influx also *via* a GPCR, most likely by affecting both L- and P/Q- Ca^2+^ channels. Surprisingly, Ca^2+^ influx was significantly reduced by a PKA blocker, indicating an involvement of PKA in hypothalamic neurons (26). The effects of nesfatin-1 in sensory neurons may be mediated through the participation of a G_i/o_ protein coupled receptor as it is speculated that the nesfatin-1-induced Ca^2+^ increase may result from a Ca^2+^ influx from both extracellular and intracellular sources (58). Furthermore, several other studies proposed an influence of nesfatin-1 on calcium levels in various tissues: Exposure of the isolated and perfused working heart to nesfatin-1 results in positive inotropism mediated through cAMP, PKA, L-type Ca^2+^ channels, sarcoplasmic/endoplasmic reticulum calcium ATPase 2a (SERCA2a) pumps, ERK1/2 and phospholamban (PLN) (53). On the other hand, it was found that nesfatin-1 leads to increased expression of Atp2a2a mRNA encoding SERCA2a, while there were no changes observed in the expression of ryanodine receptor 1b (RyR1b) encoding mRNA (55). Furthermore, a different study demonstrated that nesfatin-1 induces heart failure during clinical treatments by increasing expression of the cardiac L-type Ca^2+^ channel a1c subunit (22). Likewise, it was shown that in mice, nesfatin-1 dose-dependently stimulates intracellular Ca^2+^ levels *via* L-type Ca^2+^ channels independently of PKA and phospholipase A2 (PLA2) in beta-cells under elevated plasma glucose concentration (56). One mechanism of the elevated Ca^2+^ influx could be an increase in membrane Na^+^ permeability, which depolarizes the membrane to open voltage-gated calcium channels (VDCC) (90), a mechanism used by glucagon-like peptide 1 (GLP-1) (91). Another potential explanation is an involvement of protein kinase C, which has been reported to increase glucose-stimulated Ca^2+^ influx through VDCC and insulin secretion (92). In contrast, another study reported that peripheral nesfatin-1 stimulates Ca^2+^ influx through voltage-gated N-type channels, thereby directly activating afferent vagal neurons (41). On the other hand, nesfatin-1 showed an effect on vagal neurons by mediating Ca^2+^ signaling through T-type channels, which are low-voltage activated channels localized in different areas of the central nervous system (75, 93, 94). The ultimate role of T-type Ca^2+^ channel activation in the response of vagal neurons to nesfatin-1 and the downstream target/effect of this signaling pathway remain unknown.

However, nesfatin-1 not only regulates the calcium influx *via* various mechanisms, but also influences the levels of other ions: More precisely, nesfatin-1 directly induces an inhibition of the voltage-dependent potassium (Kv) current by directly binding to Kv2.1 channels to exert its effect on pancreatic beta cells, as these Kv channels have been identified as the major contributors to Kv currents in these cells. Since nesfatin-1 is localized in beta cells, it has been hypothesized that nesfatin-1 may affect beta cell function in an autocrine/paracrine manner (51). Another finding assumes that nesfatin-1 activates K_ATP_ channels, thereby inhibiting orexigenic neuropeptide Y (NPY) neurons of the arcuate nucleus and consequently leading to satiety (61).

#### 4.1.6 NO-cGMP System

Interestingly, nesfatin-1 also targets the NO-cGMP system; again, the particular receptor(s) is/are still unknown and require further research: In atrial tissue nesfatin-1 might increase the sensitivity of smooth muscle to cGMP-mediated relaxing mechanisms rather than increasing the NO levels. Interestingly, these positive chronotropic effects in the atrial tissue were independent of the β1-adrenergic receptor (24). On the other hand, nesfatin-1 also impairs the SNP-induced cGMP production, thereby inhibiting the NO donor-induced smooth muscle relaxations. Since these effects were persistent in the presence of IBMX, a phosphodiesterase (PDE) inhibitor, it can be suggested that these effects may be mediated *via* inhibition of soluble guanylate cyclase activity rather than activation of PDEs (16). However, other findings indicated that by recruiting guanylyl cyclase-linked receptors, namely natriuretic peptide receptor type A (NPR-A), and thereby involving the cGMP/protein kinase G pathway and ERK1/2, nesfatin-1 mediates negative inotropic and lusitropic effects in rats. Since the use of pertussistoxin did not inhibit the effects of nesfatin-1, the hypothesis that nesfatin-1 binds to a G_i/o_ cannot be confirmed by these observations (21).

#### 4.1.7 AKT Signaling Pathway

Also involved in multiple aspects of the effects of nesfatin-1 is the AKT signaling pathway: In the myocardium of mice with myocardial infection, nesfatin-1 increases expressions of phosphorylated-AKT/AKT and phosphorylated-glycogen synthase kinase 3 beta (GSK-3β)/GSK-3β, thereby protecting cardiac tissue (71). Other findings indicate that overexpression of nesfatin-1 in human trophoblasts influences the expression of phosphoinositide-3-kinase (PI3K)/AKT/mTOR and AKT/GSK3β pathway, contributing to trophoblast dysfunction simultaneously suppressing oxidative stress by reducing reactive oxygen species (ROS), malondialdehyde (MDA), and superoxide dismutase (SOD) levels (48). Furthermore, in vascular smoot muscle cells (VSMC) nesfatin-1 modulates the crosstalk between PI3K/AKT/mTOR and Janus kinase 2 (JAK2)/STAT3 signaling, leading to hypertension and vascular remodeling (50). Nesfatin-1 also dose-dependently increased MMP-2 and MMP-9 levels, while it decreased PPARγ gene expression in VSMCS contributing to vascular remodeling and neointimal hyperplasia (83). Regarding STAT3 signaling, hypothalamic nesfatin-1 in rats activates the mTOR-STAT3 pathway, thereby regulating glucose homeostasis and hepatic insulin sensitivity (74). Moreover, studies involving HL-1 cells and cardiomyocytes indicate a nesfatin-1-induced phosphorylation of ERK1/2, AKT and the substrate of AKT, AS160, thereby stimulating peripheral GLUT-4 translocation (34). These findings are in line with the results of a recent study reporting nesfatin-1 to affect glucose metabolism by affecting AKT phosphorylation and GLUT4 membrane translocation in adipose tissue, liver, and skeletal muscle (14). The specific receptor to which nesfatin-1 binds to trigger these effects is still unknown.

### 4.2 Colocalization With Other Peptides

Furthermore, PVN neurons in rats are directly depolarized by nesfatin-1, suggesting a mediation of these effects through the activation of a GPCR. Since PVN neurons respond to nesfatin-1 and further produce nesfatin-1, there might be potential interactions between various subgroups of PVN neurons engaged in the control of several autonomic outputs. The specificity of the effects may also arise from the co-expression of other receptors on nesfatin-1-sensitive neurons. An alternative explanation is that multiple nesfatin-1 receptors exist that are separately responsible for hyperpolarizing or depolarizing responses (60). It was shown that in the PVN, nesfatin-1 targets magnocellular and parvocellular Oxt neurons as well as nesfatin-1 neurons themselves, further stimulating Oxt release. Furthermore, the presence of nesfatin-1 specifically in the secretory vesicles of PVN neurons was found, indicating paracrine/autocrine actions of nesfatin-1 (52). On the other hand, it was suggested that the hypertensive effect of nesfatin-1 may require both activation of Oxt neurons and recruitment of CRF neurons (81). Moreover, by targeting ghrelin and Oxt receptors nesfatin-1 suppresses neutrophil infiltration and improves glutathione levels in acute pancreatitis (27) and colitis (59). Interestingly, the effects of nesfatin-1 on myeloperoxidase activity, lipid peroxidation and glutathione level were abrogated by application of melanocortin receptor antagonist. Despite previously demonstrating that nesfatin-1 utilizes the melanocortin signaling pathway for its effects on food intake, these findings indicate that nesfatin-1 may also utilize the receptors mediating its anorexigenic effect for anti-inflammatory effects (59). Since the effects of nesfatin-1 on GD- excitatory neurons and GD-inhibitory neurons in the PVN were attenuated in the presence of H4928, an Oxt receptor antagonist, it can be assumed that nesfatin-1involves Oxt receptors to modulate gastric function (38). Moreover, in goldfish nesfatin-1 inhibits ghrelin mRNA expression in the brain and vice versa, leading to the assumption of a negative feedback inhibition of endogenous ghrelin and nesfatin-1 (45). Another study indicates that peripheral nesfatin-1 most likely exerts part of its effect directly *via* ghrelin receptor (GHSR) signaling. Furthermore, the results here show that the effects of nesfatin-1 in mice fed a normal chow diet, such as improvement of glucose tolerance, upregulation and phosphorylation of AKT-kinase mRNA and GLUT4 membrane translocation, were dependent on the presence of GHSR. In high-fat diet (HFD) fed mice, nesfatin-1 additionally led to a raise of AKT levels in liver tissues, which is also a GHSR-dependent mechanism (33). Therefore, it is possible that nesfatin-1 acts as an endogenous inverse agonist of the GHSR and influences the structure or activity of the GHSR. Furthermore, it was found that nesfatin-1 inhibits the excitability of dopaminergic neurons in the ventral tegmental area (VTA) (28) and the substantia nigra (47), thereby decreasing dopamine release (28). In addition, nesfatin-1 signaling in the lateral hypothalamic area (LHA) or electric stimulation of the Arc modulate the activation of GD-responsive neurons, gastric motility and gastric secretion involving melanin-concentrating hormone signaling indicating the potential presence of nesfatin-1 specific receptors in these neurons (76). Recent findings also provide evidence that nesfatin-1 administered into the amygdala targets glucocorticoid and mineral corticoid receptor pathways, thereby being involved in the pathophysiology of irritable bowel syndrome (IBS)-like visceral hypersensitivity (86). Lastly, predominantly *via* the CCK-CCK1R signaling pathway nesfatin-1 reduces food intake in Siberia sturgeon (85).

Involving cholinergic pathways, central administration of nesfatin-1 elevates mean arterial pressure and modulates heart rate in rats. These cardiovascular effects were mediated by both nicotinic and muscarinic receptors. Considering that recent studies have shown that both muscarinic and nicotinic acetylcholine receptors interact with G-coupled proteins (95), it may be that nesfatin-1 mediates its cardiovascular effects *via* central muscarinic and nicotinic receptors because of its affinity to GPCRs (23).

Furthermore, it was found that central nesfatin-1 can increase peripheral sympathetic outflow and thus β-adrenergic activation, resulting in iBAT thermogenesis and body weight loss. It was demonstrated that the thermogenetic effect of nesfatin-1 mainly depends on β3-adrenergic stimulation. In addition, levels of Dio2 and cell death inducing DFFA like effector A (CIDEA) mRNA were increased in brown adipose tissue after nesfatin-1 administration, which is plausible as both are involved in regulating the thermogenic program (46). Since the direct effects of peripherally injected nesfatin-1 on vascular smooth muscle were attenuated by pretreatment with propranolol, an involvement of the β-adrenergic system can also be suspected here (57).

### 4.3 Implications for Future Research

The described various distribution of the nesfatin-1 receptor further supports the assumption that nesfatin-1 may be involved in the regulation of various homeostatic functions, and the various signaling pathways underlying the actions of nesfatin-1 emphasize its multiple effects. The recent discoveries about the diverse effects of nesfatin-1 indicate the importance of evaluating and determining the potential use of nesfatin-1 in a therapeutic context in the future. For this reason, further research is needed to fully understand the exact cascades of the peptide and its complex interplay with other hormones.

## 5 Conclusion

This present review highlights nesfatin-1 as a pleotropic peptide that acts at multiple levels in the organism, thereby eliciting a wide variety of effects. As diverse as the effects of the peptide, so are the intracellular signaling pathways and downstream effects summarized here, including the influence of nesfatin-1 on various hormones and their receptors. The identification of the so far unknown nesfatin-1 receptor will represent a major leap forward in our understanding of the physiology of nesfatin-1 and will allow us to better investigate the precise mechanisms underlying the many different effects of the peptide. Further research is needed so we might be able to consider a therapeutic use of nesfatin-1 in the future.

## Data Availability Statement

The original contributions presented in the study are included in the article/supplementary material. Further inquiries can be directed to the corresponding author.

## Author Contributions 

EW performed the systematic search. SR and EW screened the papers and SR wrote the first draft of the manuscript. AS planned the study and gave critical input throughout the study. All authors contributed to the article and approved the submitted version.

## Conflict of Interest

The authors declare that the research was conducted in the absence of any commercial or financial relationships that could be construed as a potential conflict of interest.

## Publisher’s Note

All claims expressed in this article are solely those of the authors and do not necessarily represent those of their affiliated organizations, or those of the publisher, the editors and the reviewers. Any product that may be evaluated in this article, or claim that may be made by its manufacturer, is not guaranteed or endorsed by the publisher.
